# A Preliminary Study of Three-dimensional Sonographic Measurements of the Fetus

**DOI:** 10.5041/RMMJ.10203

**Published:** 2015-04-29

**Authors:** Udi Ergaz, Israel Goldstein, Michael Divon, Zeev Weiner

**Affiliations:** 1 Department of Obstetrics and Gynecology, Rambam Medical Center, Haifa, Israel;; 2 Rappaport Faculty of Medicine, Technion–Israel Institute of Technology, Haifa, Israel;; 3 Department of Obstetrics and Gynecology, Lenox Hill hospital, New York, NY, USA

**Keywords:** Fetus, three-dimensional, ultrasound

## Abstract

**Objectives:**

This study was aimed at establishing an ideal method for performing three-dimensional measurements of the fetus in order to improve the estimation of fetal weight.

**Methods:**

The study consisted of two phases. Phase I was a prospective cross-sectional study performed between 28 and 40 weeks’ gestation. The study population (*n*=110) comprised low-risk singleton pregnancies who underwent a routine third-trimester sonographic estimation of fetal weight. The purpose of this phase was to establish normal values for the fetal abdominal and head volumes throughout the third trimester. Phase II was a prospective study that included patients admitted for an elective cesarean section or for induction of labor between 38 and 41 weeks’ gestation (*n*=91). This phase of the study compared the actual birth weight to two- (2D) and three-dimensional (3D) measurements of the fetus. Conventional 2D ultrasound fetal biometry was performed measuring the biparietal diameter (BPD), head circumference (HC), abdominal circumference (AC), and femur diaphysis length (FL). Volume estimates were computed utilizing Virtual Organ Computer-aided AnaLysis (VOCAL), and the correlation between measured volumes and actual neonatal weight was calculated.

**Results:**

Overall, this longitudinal study consisted of 110 patients between 28 and 41 weeks’ gestation. Normal values were computed for the fetal abdomen and head volume throughout the third trimester. Ultrasound examination was performed within three days prior to delivery on 91 patients. A good correlation was found between birth weight and abdominal volume (*r*=0.77) and between birth weight and head volume (*r*=0.5). Correlation between bidimensional measurements and actual fetal weights was found to be comparable with previously published correlations.

**Conclusion:**

Volume measurements of the fetus may improve the accuracy of estimating fetal size. Additional studies using different volume measurement of the fetus are necessary.

## INTRODUCTION

Fetal measurements obtained by prenatal ultra-sonography have become an integral part of fetal assessment. They are used for estimating fetal weight and for measuring fetal organs. Fetal weight estimation is obviously important for recognizing intrauterine growth restriction and macrosomia, both of which require planning the time and mode of delivery. Measurements of fetal organs are also important for diagnosing fetal abnormalities such as microcephaly and skeletal abnormalities. Therefore, different algorithms and tables have been established in the past for estimating fetal weight and for creating nomograms for fetal organs size throughout gestation.^1^,^2^ However, the traditional methods for these measurements were based on the use of two-dimensional (2D) ultrasound. For example, even though the fetal body is a voluminous mass, its weight is traditionally calculated by using only two dimensions, with a 10%–15% deviation.

We hypothesized that by using three-dimensional (3D) ultrasound we would be able to improve the accuracy of fetal measurements as well as the estimation of fetal weight. To that end, we initiated this preliminary study to determine the ideal method for performing 3D measurements of the fetal abdomen and head. This paper presents the results of that study.

## METHODS

The study was performed in the Division of Ultrasound in Obstetrics and Gynecology at Rambam Medical Center, Haifa, Israel between January 2011 and July 2012. The study consisted of two phases and two different study populations, respectively.

### Phase I

Phase I was aimed at establishing the normal values for fetal abdominal and head volumes throughout the third trimester of pregnancy. A prospective cross-sectional study was performed between 28 and 40 weeks of gestation. All patients included in the study had low-risk singleton pregnancies; each patient underwent a routine third-trimester sonogram to estimate fetal weight.

### Phase II

Phase II was a prospective study that included patients admitted for an elective cesarean section or for induction of labor between 38 and 41 weeks of gestation.

This phase of the study compared the actual birth weights with the estimated 2D and 3D fetal measurements.

The criteria for participating in the study included: well-defined gestational age based on embryonic/fetal crown–rump length measurement during the first trimester; normal fetal anatomy scans; delivery within three days of acquisition of the 2D measurements and 3D volumes. The study was approved by the Institutional Review Board, and all participating patients signed an informed consent.

Maternal age, gestational age, and parity were recorded at the time of the scan. The subjects included in this study were mostly Caucasians from all socioeconomic backgrounds. Data on the gestational age at birth, mode of delivery, and clinical characteristics of the newborn were collected post-partum from the hospital records of the mother and the neonates. All neonates were weighed immediately after birth in the delivery room.

### Equipment Used for the Studies

Ultrasound examinations were performed using a Voluson 730 Pro (GE Healthcare, Solingen, Germany) machine using a RAB 4–8L probe. All ultrasound examinations were performed transabdominally by two physicians (U.E. and Z.W.).

### Two-dimensional Ultrasound Measurements

Conventional 2D ultrasound fetal biometry was performed as follows: Head measurements were obtained in the axial view at the level of the cavum septi pellucidi, where both thalami could be seen symmetrically and the anterior and posterior aspects of the cerebral falx were equidistant to the parietal bones. The biparietal diameter (BPD) was measured from the outer edge of the proximal parietal bone to the inner edge of the distal skull table, in a line perpendicular to the orientation of the cerebral falx. The head circumference (HC) was calculated using the scanner’s automatically generated ellipse including the outer margins of the fetal skull. Abdominal circumference (AC) was measured using the scanner’s automatically generated ellipse on a transverse circular view of the abdomen at the level of the stomach and the porto-umbilical vein complex. Femur diaphysis length (FL) was measured in a plane in which the full femoral diaphysis was almost parallel to the transducer surface, and the measurement was taken from one end of the diaphysis to the other.

### Three-dimensional Ultrasound Measurements

Acquisition and storage of 3D data sets of the fetal head and abdomen were performed as follows: Initially, the transducer was held over the planes as described above for the BPD and AC 2D acquisitions.

**Figure 1.**
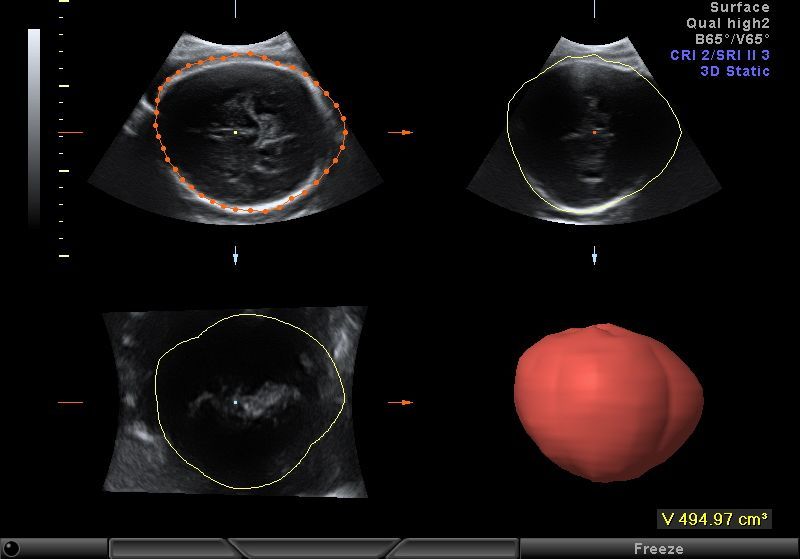
Plans of the Fetal Head Used for Three-dimensional Reconstruction.

**Figure 2.**
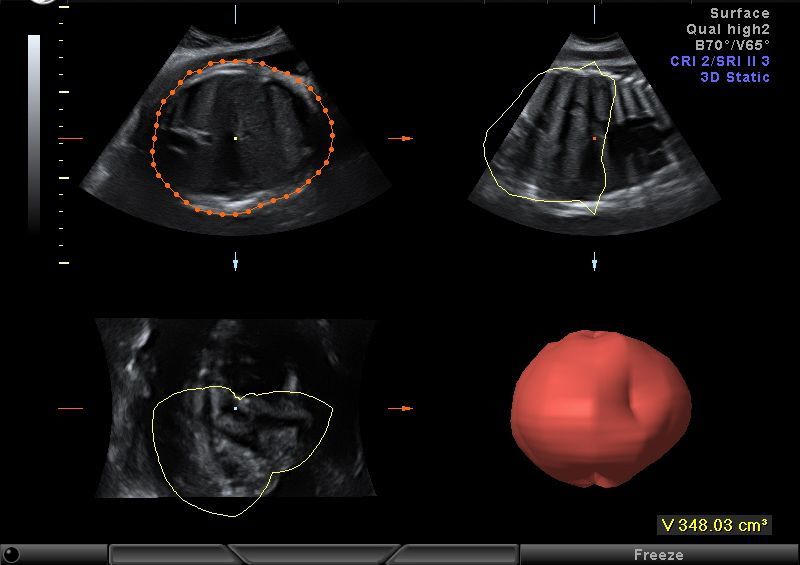
Plans of the Fetal Abdomen Used for Three-dimensional Reconstruction.

Volumes were acquired using automatic sweeps; the sweep angle was set at 30°. The acquisition process was repeated if there was any maternal or fetal movement. Head and abdomen volume acquisition by the VOCAL technique was performed as follows: the data set containing the fetal head or abdomen was displayed on the screen in the transverse view, and this image was rotated so that the head or the abdomen was identified in a perpendicular position. Volume estimates were computed using the Virtual Organ Computer-aided AnaLysis (VOCAL) program version 5.3 (GE Medical Systems, Solingen, Germany) with a manual trace at 30° of rotation, so six planes were demonstrated. Traces of the scanned organ contours were performed manually using a touch screen stylus pen directly on the displayed image. Figures 1 and 2 present the head and abdominal plans used for reconstructing the 3D images. Fetal abdominal volume was measured between the fetal diaphragm and pelvis. Fetal head volume was measured above the base of the skull.

### Statistical Analysis

Normal values for the abdomen and head volumes were calculated throughout the third trimester of pregnancy. Pearson’s correlation was used to compare between the 2D and 3D measurements and the birth weights.

## RESULTS

### Phase I

A total of 110 patients participated in the longitudinal study between 28 and 41 weeks of gestation. Three patients who developed intrauterine growth restriction and three patients who developed gestational diabetes were excluded from the study. The mean maternal age of this study group was 30.45±4.9 years; 55% were primiparous. Mean birth weight was 3498.7±480 g, and mean gestational age at delivery was 40.23±1.3 weeks. The normal values calculated for the fetal abdomen and head volumes are presented in Table 1.

**Table 1.** Normal Values of Fetal Abdominal and Head Volume throughout the Third Trimester.

**Weeks of Gestation**	**Abdominal Volume (mm^3^)**	**Head Volume (mm^3^)**
28–30	249.5±30	328.8±50.6
30–32	433±29.4	435.2±34.4
32–34	464±30.3	455.8±39.6
34–36	496.5±44.4	482.8±44
36–38	521.3±39.8	501.5±46.7
38–40	539.5±40.1	512.5±41.3

### Phase II

A total of 91 patients had ultrasound examination performed within three days prior to delivery. The mean maternal age of this study group was 29.65±3.9 y; 25% were primiparous. Mean birth weight was 3454.7±440 g, and mean gestational age at delivery was 39.33±1.3 weeks. Correlations between 2D and 3D measurements of the fetus and birth weight are presented in Table 2. As shown in this table, similar results were obtained using 2D or 3D measurements.

**Table 2.** Correlation between Birth Weight and 2D and 3D Fetal Measurements.

	**Abdominal Volume**	**Head Volume**	**Abdominal Circumference**	**Biparietal Diameter**	**Head Circumference**	**2D Estimated Fetal Weight**
Birth Weight	0.77*	0.5*	0.81*	0.419*	0.545*	0.793*

* p<0.0001

## DISCUSSION

Prenatal 3D ultrasound has been widely used during the last decade for different purposes. Fetal volume measurements have been studied in the first trimester of pregnancy suggesting that a small fetal volume may result in earlier detection of high-risk pregnancies.^2^–^4^ During the second trimester, 3D ultrasound appeared to be valuable in the anatomical survey of the fetus.^5^–^7^ There has been a clear benefit in using 3D ultrasound for detection of clinical situations such as facial cleft, brain anomalies, and spinal defects. One of the most studied fields using fetal 3D ultrasound has been fetal echocardiography.^8^ Finally, volume measurements have been also used for estimating the amniotic fluid volume and placental size.^5^

Estimation of fetal size is one of the most important goals in prenatal diagnosis. Prenatal diagnosis of intrauterine growth restriction allows early intervention and improvement of pregnancy outcome, while prenatal diagnosis of fetal macrosomia may avoid birth trauma. However, estimation of fetal weight based on the existing formulas is still limited, especially in macrosomic fetuses.^2^,^9^ Hoping that 3D fetal measurements would improve estimation of fetal size, we initially focused on studying the fetal abdominal and head volume. There are few publications attempting to describe fetal measurements via 3D techniques. Bromley et al. used offline 3D reconstruction of the third- trimester fetus. The authors concluded that this technique is a reliable method for estimating fetal weight.^10^ Yang et al. have shown that the use of 3D ultrasound compared to 2D, even by an inexperienced operator, allows faster measurements of the fetus.^11^ Nardozza et al. performed 3D measurements of the fetal upper arm and thigh and created formulas to predict birth weight. The authors concluded that the new formulas, based on 3D measurements, were not superior to 2D formulas.^12^

We have used rotational measurements of volume using the VOCAL imaging program, which extends the 3D view. This technique allows rotation of the 3D data set around a central axis through a number of rotation steps. Volume calculation in the *in vitro* setting has been proved reliable and valid to within 4% of the “actual” volume.^13^ It is noteworthy that comparison between contemporaneous sonographic and 3D magnetic resonance at late gestational age demonstrated an acceptable correlation between the two techniques for fetal head and abdomen measurements.^14^ Our results in the Phase II study demonstrated a similar correlation with birth weight when comparing the conventional 2D and the 3D sonograms. However, the fetal abdomen and head volume measurements were not superior to the traditional 2D measurements of abdominal and head circumference, and biparietal diameter.

In conclusion, fetal volume measurements may improve the accuracy of fetal size estimations. Future studies should use different volume measurements, which may improve the accuracy.

## Abbreviations:

2D: two-dimensional;
3D: three-dimensional;
AC: abdominal circumference;
BPD: biparietal diameter;
FL: femur diaphysis length;
HC: head circumference;
VOCAL: Virtual Organ Computer-aided AnaLysis.
